# Bioinformatic analysis of the human *DHRS4 *gene cluster and a proposed mechanism for its transcriptional regulation

**DOI:** 10.1186/1471-2199-11-43

**Published:** 2010-06-03

**Authors:** Zhong-Jing Su, Qiao-Xia Zhang, Ge-Fei Liu, Xu-Hong Song, Qi Li, Rui-Jian Wang, Hai-Bin Chen, Xiao-Yuan Xu, Xu-Xia Sui, Dong-Yang Huang

**Affiliations:** 1 Department of Cell Biology, 22 Xinling Road, Shantou University Medical College, Shantou, Guangdong, 515041, China; 2 Department of Histology and Embryology, 22 Xinling Road, Shantou University Medical College, Shantou, Guangdong, 515041, China

## Abstract

**Background:**

The human *DHRS4 *gene cluster consists of three genes, *DHRS4*, *DHRS4L2 *and *DHRS4L1*. Among them, *DHRS4 *encodes NADP(H)-dependent retinol dehydrogenase/reductase. In a previous study, we investigated the alternative splicing of *DHRS4 *and *DHRS4L2*. *DHRS4L1 *was added to the gene cluster recently, but little is known about its structure and expression. To reveal the regulatory mechanism of the *DHRS4 *gene cluster expression, we studied the structure and transcription of *DHRS4L1 *in the context of the transcriptional behaviors of the human *DHRS4 *gene cluster. Based on the results of bioinformatics analysis, we propose a possible mechanism for the transcriptional regulation of the human *DHRS4 *gene cluster.

**Results:**

The homologous comparison analysis suggests that *DHRS4*, *DHRS4L2 *and *DHRS4L1 *are three homologous genes in human. *DHRS4L1 *and *DHRS4L2 *are paralogues of *DHRS4*, and *DHRS4L2 *is the most recent member of the *DHRS4 *gene cluster. In the minus strand of the human *DHRS4 *gene cluster, a gene transcribed in an antisense direction was found containing a 5' sequence overlapping the region of exon 1 and promoter of *DHRS4*. By cloning the full length of RNA variants through 5'RACE and 3'RACE, we identified two transcription start sites, within exon *a2 *and exon 1, of this newly named gene *DHRS4L1 *using neuroblastoma cell line BE(2)-M17. Analysis of exon composition in the transcripts of *DHRS4 *gene cluster revealed that exon 1 was absent in all the transcripts initiated from exon *a1 *of *DHRS4L2 *and exon *a2 *of *DHRS4L1*.

**Conclusions:**

Alternatively spliced RNA variants are prevalent in the human *DHRS4 *gene cluster. Based on the analysis of gene transcripts and bioinformatic prediction, we propose here that antisense transcription may be involved in the transcriptional initiation regulation of *DHRS4 *and in the posttranscriptional splicing of *DHRS4L2 *and *DRHS4L1 *for the homologous identity of *DHRS4 *gene cluster. Beside the alternative transcriptional start sites, the antisense RNA is novel possible factor serving to remove exon 1 from the transcripts initiated from exon *a1 *and exon *a2*.

## Background

*DHRS4*, the fourth member of the dehydrogenase/reductase (SDR) family, is a gene encoding NADP(H)-dependent retinol dehydrogenase/reductase (NRDR). Prior to December of 2008, an examination of the *DHRS4 *gene on chromosome 14q11.2 in GenBank revealed that the human *DHRS4 *gene cluster consisted of two genes, *DHRS4 *and *DHRS4L2*. In 1997, we identified the NRDR proteins from rabbit liver tissues, and found that this protein had strong retinol oxidation and retinal reduction activities and was a crucial enzyme in the metabolism and synthesis of retinoic acid [1], an important intracellular signaling molecule involved in the regulation of cell growth, differentiation of embryonic cells, and the regulation of immune functions. The presence of NRDR in other species was confirmed recently and the enzyme was shown to have similar carbonyl reductase activity but with different substrates [2-4]. In previous studies, we also found the alternative spliced RNAs of *DHRS4 *and *DHRS4L2 *[5-7], and identified exon *a1 *both as a novel exon contained in the RNA variants of *DHRS4L2 *and an alternative transcription start site (TSS) for *DHRS4L2 *[7]. Exon *a1 *matches with a special intergene sequence, approximate 19 kb upstream of the first exon of *DHRS4L2 *and 559 bp downstream of the last exon of *DHRS4 *in the genomic DNA.

Due to an information update of GenBank in December of 2008, the gene *LOC728635*, located downstream of *DHRS4 *and *DHRS4L2 *and similar to the peroxisomal short-chain alcohol dehydrogenase, was renamed *DHRS4L1*. The human *DHRS4 *gene cluster is now composed of *DHRS4*, *DHRS4L2 *and *DHRS4L1*. To investigate the transcription of *DHRS4L1 *and the transcriptional regulation of *DHRS4 *gene cluster, we cloned the full length RNA variants of *DHRS4L1 *in this study, and found that *DHRS4L1 *initiated its transcription from two TSS, exon *a2 *and exon 1. Exon *a2 *matches with the intergene sequence between *DHRS4L2 *and *DHRS4L1*, and is homologous to exon *a1 *located upstream of *DHRS4L2*. Through the analysis of the gene structures and transcriptional behaviors of the *DHRS4 *gene cluster, we propose a possible mechanism involving the antisense transcripts from *C14orf167 *and the alternative TSS for the transcriptional regulation of the human *DHRS4 *gene cluster.

## Results and discussion

### Comparative analysis of the *DHRS4 *gene cluster

**Figure 1:**
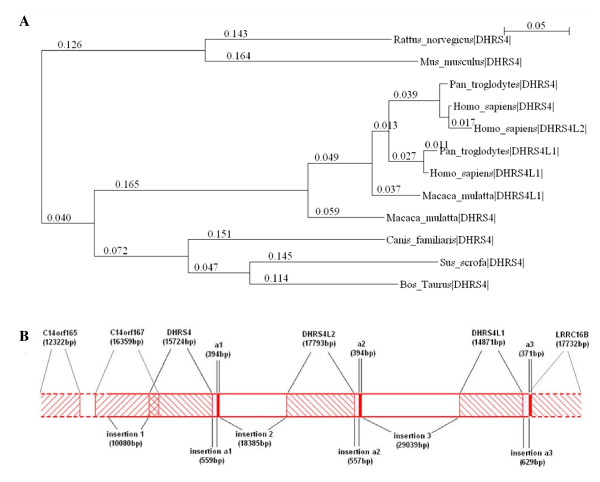
**The homologous comparison of *DHRS4 *gene cluster**. (A) Evolutionary relationships of *DHRS4*, *DHRS4L2 *and *DHRS4L1 *among various species. The phylogeny of *DHRS4*, *DHRS4L2 *and *DHRS4L1 *is shown as a neighbor-joining tree with bootstrap. The scale bar represents 5% sequence divergence. Positions of genes in the corresponding species are shown as vertical hatches. Horizontal bars and the values on them represent the length of the DNA sequence after alignment. (B) The context and composition of the human *DHRS4 *gene clusters.

In the three copies of the *DHRS4 *gene cluster, *DHRS4 *is common in mammals and aquatic animals, while *DHRS4L1 *is only found in primates and *DHRS4L2 *in humans. Humans, therefore, are the only species that contain all three genes in the *DHRS4 *gene cluster. The phylogenic tree of the *DHRS4 *gene cluster across different species (Figure 1A) and comparative analysis of the human *DHRS4 *gene cluster (Table 1 and Table 2) suggest that *DHRS4L2 *and *DHRS4L1 *are paralogues of *DHRS4*, and that *DHRS4L2 *is the most recent member of the *DHRS4 *gene cluster. The gene sequence identity between *DHRS4L2 *and *DHRS4L1 *is lower than that of *DHRS4L2 *and *DHRS4*, indicating that *DHRS4L2 *was duplicated directly from *DHRS4*, not from *DHRS4L1*.

**Table 1:** Homologous comparison of the human gene *DHRS4*, *DHRS4L2 *and *DHRS4L1*.

	DNA				cDNA
		
	Insertion*	Gene*	Insertion *a*	Exon *a*	
*DHRS4 vs DHRS4L2*	62.0%	97.5%	98.7%	98.9%	90.9%
*DHRS4 vs DHRS4L1*	38.0%	77.8%	70.5%	78.9%	87.4%
*DHRS4L2 vs DHRS4L1*	42.9%	77.7%	70.3%	79.2%	81.7%

* Long repeat sequences were removed before comparison.

**Table 2:** Exons composition and the homologous relationship of them among the *DHRS4 *gene cluster.

*	Exon 1	Exon 2	Exon 3			Exon 4	Exon 5	Exon 6	Exon 7	Exon 8
*DHRS4*	161	178	102			71	52	135	56	534
*DHRS4L2*	258	178	102			71	52	134	56	531
**	Exon 1	Exon 2	Exon 3	Exon 4	Exon 5	Exon 6	Exon 7	Exon 8	Exon 9	Exon 10
*DHRS4L1*	128	178	54	50	7	71	52	135	56	518

* corresponding exon number of *DHRS4*, *DHRS4L2*, ** corresponding exon number of *DHRS4L1*

In our previous study, a novel exon *a1 *was identified at the 5'end of the *DHRS4L2 *transcripts, and the sequence of exon *a1 *matches with the intergene sequence between *DHRS4 *and *DHRS4L2 *in genomic DNA [7]. Through sequence BLASTing, two other homologous sequences of exon *a1 *were found and each was localized downstream of the *DHRS4L2 *or *DHRS4L1 *sequences. In this study, we named these three homologous sequence of exon *a *as exon *a1*, exon *a2 *and exon *a3*, each located downstream of *DHRS4*, *DHRS4L2 *and *DHRS4L1 *respectively. The spaces between an exon *a *and its upstream gene was named as insertion *a1*, insertion *a2 *and insertion *a3 *while the spaces between exon *a *and its downstream gene was named as insertion 1, insertion 2 and insertion 3, respectively (Figure 1B). The analysis of the gene structure and homology of the *DHRS4 *gene cluster suggested that *DHRS4 *duplication was initiated from the "insertion," leading to the formation of a copy unit, "insertion-*DHRS4 *gene-insertion *a*-exon *a*", which was copied twice in chromosome 14 (14q11.2) with the resultant formation of the *DHRS4 *gene cluster (Figure 1B). Genome duplications are considered as the results of long-term biological evolution and accounting for the bulkiness and complexity of the human genome [8,9]. Through the comparative analysis, we identified the general structure of *DHRS4 *gene cluster, and designed the following assays to provide insights into the transcriptional regulation of this gene cluster.

### Transcription of the human *DHRS4 *gene cluster

The reference sequences of the human *DHRS4 *and *DHRS4L2 *cDNA are 1289 bp and 1382 bp respectively, each consisting of 8 exons. *DHRS4L1 *has a full length of 1249 bp and is composed of 10 exons (Table 2). Based on analysis of the RNA variants of *DHRS4 *and *DHRS4L2 *identified in our previous studies and related data from GenBank, we found that the alternative splicing was prevalent in *DHRS4 *and *DHRS4L2 *(Table 3), moreover, *DHRS4L2 *harbored two alternative TSS (exon *a1 *and exon 1), while *DHRS4 *initiated its transcription only from one site (exon 1). To explore the transcriptional regulation mechanism of the human *DHRS4 *gene cluster, we first tested the presence of the *DHRS4L1 *RNA variants in this study since little was known about the transcription of *DHRS4L1*.

**Table 3:** Alternative splicing variants of the human *DHRS4 *gene cluster.

*DHRS4*		*DHRS4L2*				*DHRS4L1*	
**No**.	**Exon**	**No**.	**Exon**	**No**.	**Exon**	**No**.	**Exon**

NM_021004	1-2-3-4-5-6-7-8	NM_198083	1-2-3-4-5-6-7-8			NM_0010824	1-2-3-4-5-6-7-8-9-10
AB045131	1-2-3-4-5-6-7-8	AK301373	1-2-6-7	AY616183	*a1*-2-3-7-8	AA255746	8-9-10
AF044127	1-2-3-4-5-6-7-8	BC000663	1-2-3-4-5-6-7-8	AY943857	*a1*-2-7-8	AV762338	1-8-9-10
AI087304	3-7-8	BC006125	1-2-3-4-5-6-7-8	CD244539	*a1*-2-3-4-5-6	BC171914	1-8-9-10
AK308436	1-2-3-7	BC101812	1-2-3-4-5-6-7-8	DN237879	*a1*-2	BC171918	1-8-9-10
AK314448	1-2-3-4-5-6-7-8	BC101814	1-2-3-4-5-6-7-8	DN237881	*a1*-2-3-6-7-8	BX117130	1-2
AY071856	1-2-3-7-8	DB449220	1-2	DN237882	*a1*-2-3-7-8	GQ871921	*a2*-8-9-10
AY358638	1-2-3-4-5-6-7-8	DN237888	2-3-4-5-6-7	DN237883	*a1*-2-3-7-8	GQ871922	1-8-9-10
AY616182	1-2-3-5-7-8	DN237893	2-4-5-6-7	DN237884	*a1*-2-3	GQ871923	1-8-9-10
BC003019	1-2-3-4-5-6-7-8	DN237895	2-3-5	DN237885	*a1*-2-3-5	GQ871924	*a2*-2-8-9-10
BU529016	1-2-3-7-8	DN237896	2-3-7-8	DN237886	*a1*-2-3-4-5-6-7	GQ871925	*a2*-8-9-10
DN237893	2-3-4-5-6-7-8	BQ030242	8	DN237887	*a1*-2-3-5	GQ871926	1-9-10
DQ325464	1-2-4-5-6-7-8	DQ088987	3-4-5-6-7-8	DN237890	*a1*-2-3-4-5	GQ871927	10
DQ338571	1-2-4-5-7-8	DQ088988	3-4-5-6-8	DN237891	*a1*-2-3-5		
DQ344810	1-2-3-4-5-6-7-8			DN237892	*a1*-2-4-5		

**Figure 2:**
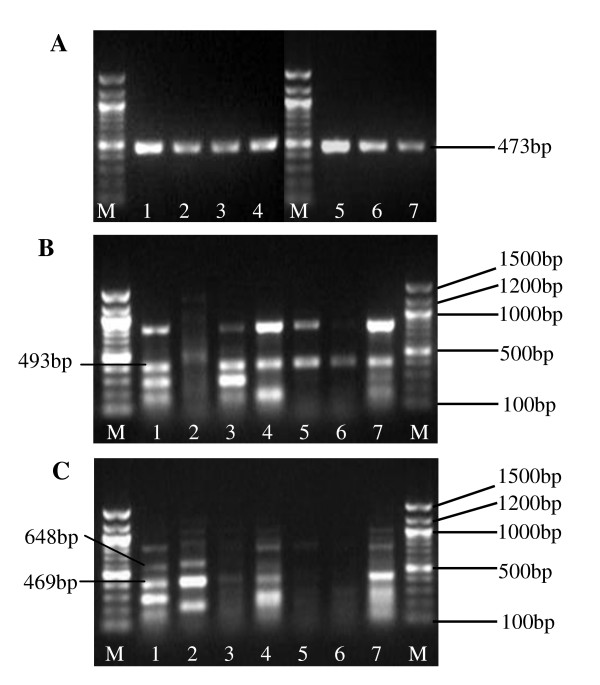
**Transcription of *DHRS4L1***. Lane 1. BE(2)-M17, 2. SK-SY5Y, 3. SK-N-SH, 4. HeLa, 5. Eca-109, 6. Hep G2, 7. HL-7702, M. marker(100 bp DNA ladder). (A) β-actin shown as a control of RT-PCR. (B) RT-PCR amplifying the exon 1-exon 10 of *DHRS4L1*. Product of 493 bp is composed of exon 1-part of intron 2-exon 8-exon 9-exon 10. (C) RT-PCR amplifying the exon *a*2-exon 10 of *DHRS4L1*. Product of 469 bp is composed of exon *a2*- exon 8-exon 9-exon 10. Product of 648 bp is composed of exon *a2*-part of insertion 3-exon 8-exon 9-exon 10.

Through 5'RACE, 3'RACE and RT-PCR (Figure 2), three full length and four partial RNA sequences of *DHRS4L1 *were amplified and identified in human neuroblastoma cell line BE(2)-M17. The sequences of these novel transcript variants were submitted to NCBI and recorded as GQ871921, GQ871922, GQ871923, GQ871924, GQ871925, GQ871926 and GQ871927. The analysis of the full length RNA variants indicates that *DHRS4L1 *starts its transcription from two alternative sites, exon *a2 *and exon 1. This suggests that the sequence upstream exon *a2 *has the potential promoter activity, similar to exon *a1*, to initiate the transcription of *DHRS4L1*. Exon *a3*, the homologous sequence of exon *a1 *downstream of *DRHS4 *and *exon a2 *downstream of *DHRS4L2*, is located downstream of *DHRS4L1 *and only 629bp away from the last exon of *DHRS4L1*, while the mRNA sequences in GenBank indicates that exon *a3 *is within the first exon of the *LRRC16B *(leucine rich repeat containing 16B) gene not related to *DHRS4 *gene cluster.

Alternative splicing patterns often show specificity related to tissue types, development stages and physiological conditions of the cells [10]. It is evident in nerve tissue that alternative splicing is correlated with the complex functions of the brain [11,12]. The results of RT-PCR to amplify the RNAs from exon 1 to exon 10 and exon *a2 *to exon 10 of *DHRS4L1 *showed that the *DHRS4L1 *RNA isoforms existed not only in cell lines from the nervous system, but also in cells from the cervical, hepatic and esophageal tissues, while different first exon (exon *a2 *and exon 1) may be utilized to initiate the transcription of *DHRS4L1 *among these cell lines (Figure 2).

Through analyzing the homologous identity of DNA and the exon composition in the transcripts variants of the *DHRS4 *gene cluster, we found that exon 1 was absent in all the spliced variants of *DHRS4L2 *and *DHRS4L1 *initiated from exon *a1 *and exon *a2 *respectively (Table 3). To verify if the absence of exon 1 is just one example found accidentally in cloned sequences or true for all the transcripts of *DHRS4 *cluster, we used forward primer in exon *a *(completely matching the sequences of exon *a1 *and exon *a2*) and reverse primer in exon 1 of *DHRS4L2 *and *DHRS4L1 *respectively to perform RT-PCR in the human neuroblastoma cell lines BE(2)-M17 and cervical carcinoma cell line HeLa. No amplifications was observed (data not shown), suggesting that exons *a1*/*a2 *and exon 1 were mutually exclusive.

In humans, majority of genes are alternatively spliced to express multiple proteins [13]. The analysis of the predicted proteins based on different *DHRS4 *RNA isoforms showed that most of them contained a domain centre of the short-chain dehydrogenase/reductase(ADH-short) family signature, possibly pointing to the functional conservation of the spliced variants. In our previous study, only one protein isoform of DHRS4 was identified and found to be correlated with the tumorigenesis of the cervical epithelia [5], while most of the spliced RNA isoforms of the *DHRS4 *gene cluster lacked an obvious open reading frame. Moreover, the full length RNA sequences of *DHRS4L2 *in the previous study [7] and that of *DHRS4L1 *in present study were investigated using the software RNAStructure4.5. This analysis predicted that low minimum free energy was required for them to form a double-stranded RNA structure. Considering the potential to form a stable secondary RNA structure and the lack of open reading frame, we speculate that most RNA variants of the *DHRS4 *gene cluster might function as non-coding RNAs to regulate gene expression.

### Transcriptional regulation of the *DHRS4 *gene cluster

Focusing our work on the alternative splicing of the *DHRS4 *gene cluster and the roles of these alternatively spliced RNAs, we have also been interested in alternative transcriptional regulation of the *DHRS4 *gene cluster. *DHRS4L2 *and *DHRS4L1 *initiate their transcription from two alternative TSS, and exon 1 is absent in all the transcripts initiated from the exons *a1 *and *a2 *of *DHSR4L2 *and *DHSR4L1 *respectively. Previous studies reported that alternative promoter was accompanied by alternative splicing of the initial exon [14,15]. Analysis of the sequence upward the exon 1 of *DHRS4L2 *and *DHRS4L1 *indicates that exon 1 lacks a canonical splice site of AG at its 5' end. It seems that the transcripts initiated from exon *a1*/*a2 *is spliced directly to the next available splice acceptor site, resulting in the removal of exon 1.

**Figure 3:**
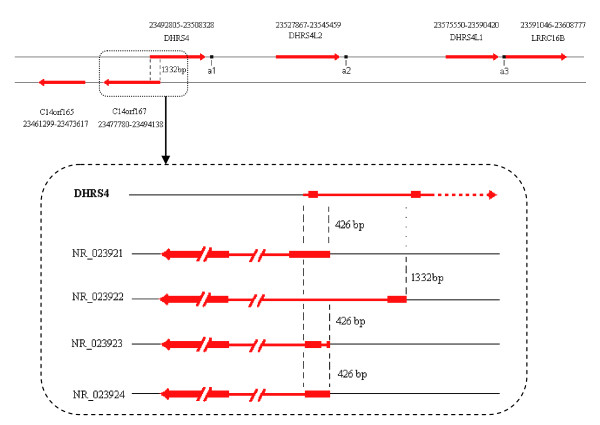
**The antisense gene *C14orf167 *and its relationship to *DHRS4***.

**Figure 4:**
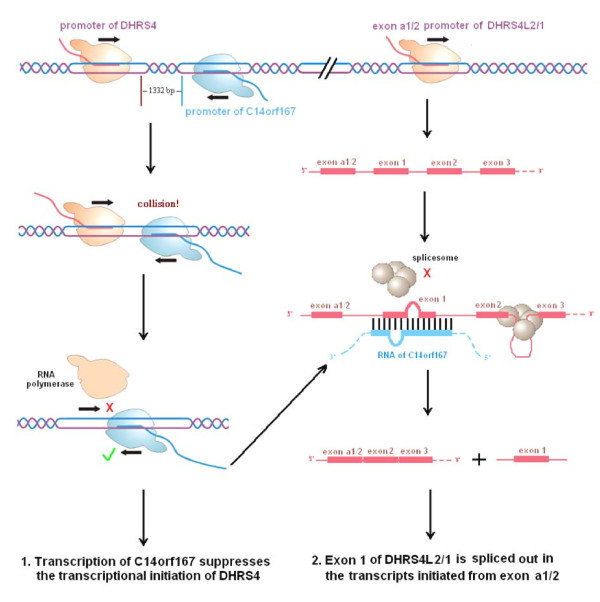
**The effects of antisense transcription from *C14orf167 *on the transcriptional regulation of the *DHRS4 *gene cluster**. (1) Bidirectional transcription of *C14orf167 *inhibits the initiation of the *DHRS4 *transcription through impeding the binding of RNA polymerase II and transcriptional factors to *DHRS4*. (2) The antisense RNAs from *C14orf167 *match with exon 1 in the primary transcripts initiated from exon *a1/a2*, and possibly resulting in the removing of exon 1 in the spliced RNAs. *DHRS4L2 *and *DHRS4L1 *are under the same condition to *C14orf167 *RNAs.

In addition, the homologous comparison of the *DHRS4 *gene cluster and analysis of the positional relationship of *DHRS4 *with the antisense gene *C14orf167 *indicate a possible mechanism for the transcriptional regulation of the *DHRS4 *gene cluster via an antisense transcribed gene. Firstly, *C14orf167 *is a typical naturally occurring antisense gene to *DHRS4*. These two genes have overlapped promoters and 5' 1332 bp coding regions (Figure 3) that belongs to the *cis *head-to-head bidirectional transcription [16-19]. Given such a positional relationship in the 5' sequence, we propose that *DHRS4 *and *C14orf167 *will interfere with each other in transcription initiation (Figure 4). Secondly, antisense RNAs may also participate in the transcriptional regulation of the trans-encoded RNAs transcribed from different loci [20,21]. Previous studies suggest that antisense RNA sequences matching with exon-intron border of the primary transcripts may prevent the binding of the splicesome to the splice site, and consequently affecting the posttranscriptional splicing [22-24]. Due to the homologies within the *DHRS4 *gene cluster (Table 1 and Table 2), the transcripts of *C14orf167 *match with exon 1 in the primary transcripts of *DHRS4L2 *or *DHRS4L1*, and possibly take part in the splicing out of exon 1 from the transcripts initiated from exon *a1*/*a2 *(Figure 4). Although the alternative promoter may affect the splicing of RNA as described above, several subclass models of the splice sites were reported in mammalian pre-mRNAs and they functioned more frequently on the first or second intron than on the other order intron [25-28]. The sequence at the 5'end of *DHRS4L2 *exon 1 is consistent with the submodel splice acceptor AC, instead of the typical AG sequence. AC functioned as a real splice acceptor site in our previous identified RNA variants (DN237887) of *DHRS4L2*. If the splice acceptor sequence at the 5'end of exon 1 in the *DHRS4 *gene cluster is efficient, the masking by antisense RNAs is a likely mechanism to explain the removal of exon 1 from the transcripts initiated from exon *a1 *or exon *a2*, although it needs further study to verify.

## Conclusions

The human *DHRS4 *gene cluster consists of *DHRS4*, *DHRS4L2 *and *DHRS4L1*. *DHRS4L2 *and *DHRS4L1 *initiate their transcription from two transcription start sites. Furthermore, exon 1 is absent in the transcripts initiated from alternative transcription start sites exon *a1 *of *DHRS4L2 *or exon *a2 *of *DHRS4L1*. Based on the analysis of the sequence relationship between *C14orf167 *and *DHRS4*, we propose that transcriptional process of *C14orf167 *may affect the transcriptional initiation of *DHRS4*. Given the homologous identities of genes within *DHRS4 *gene cluster, antisense RNAs of *C14orf167 *may take part in the posttranscriptional splicing regulation of *DHRS4L2 *and *DHRS4L1 *through masking the splicing sites and removing of exon 1 from RNA transcripts. This is a rare example of one antisense transcript regulating the transcription of different gene in *cis *and *trans *manner simultaneously, though antisense RNA and bidirectional transcription have been the focus of extensive studies recently.

## Methods

### Databases and software

The DNA, cDNA and EST sequences of the *DHRS4 *gene cluster were retrieved from the NCBI GenBank http://www.ncbi.nlm.nih.gov and UCSC Genome Browser http://www.genome.ucsc.edu. Jellyfish3.2 http://www.jellyfishsoftware.com and BLAST online http://blast.ncbi.nlm.nih.gov were employed for analyzing the homologous identity of the human *DHRS4*, *DHRS4L2 *and *DHRS4L1*. The sequences of the *DHRS4 *gene cluster in different species were aligned with Clustal X1.83 http://www.clustal.org/ and all the positions containing gaps were eliminated. A bootstrapped biological phylogenic tree was constructed using MEGA4 with the neighbor-joining method. The RNA secondary structures were predicted and analyzed with RNAstructure4.5 http://rna.urmc.rochester.edu/rnastructure.html.

### Cell culture and RNA extraction

Human neuroblastoma cell lines BE(2)-M17, SK-N-SH, SH-SY5Y, human cervical carcinoma cell line HeLa, human esophageal carcinoma cell line Eca-109, human hepatocarcinoma cell line Hep G2 and human hepatocyte cell line HL-7702 were all obtained from the Cell Bank of Chinese Academy of Sciences (Shanghai, China). The cell lines were maintained in DMEM medium supplemented with 10% (v/v) fetal bovine serum in a humidified 37°C incubator with 5% CO_2_. Total RNAs of the cell lines were extracted with TRIzol^® ^reagent (Invitrogen) and RNA quality was examined by OD260/OD280 and RNA electrophoresis.

### 5'RACE, 3'RACE and RT-PCR

The 5'end amplification of *DHRS4L1 *splicing variants in the human neuroblastoma cell line BE(2)-M17 was carried out with SMARTer™ RACE kit (Clontech), which generated a complete cDNA copy of the original mRNAs with the additional SMARTer sequence at the 5'end by the joint action of the SMARTer II A oligonucleotide and SMARTScribe reverse transcriptase. It was then used to amplify 5'end of *DHRS4L1 *through 5'RACE touchdown PCR.

The 3'end amplification was carried out using the 3'RACE system (Takara), which depended on the poly A to ligate the adaptor on the 3'end of mRNAs from the neuroblastoma cell line BE(2)-M17 for the reverse transcription into cDNA and subsequent amplification of the 3'end of *DHRS4L1 *through 3'RACE nest PCR.

RT-PCR of *DHRS4L1 *in cell lines BE(2)-M17, SK-N-SH, SH-SY5Y, HeLa, Hep G2, Eca-109 and HL-7702 were performed using Platinum *Taq *DNA Polymerase mixture (Invitrogen) after the RNA samples were reverse transcribed into cDNA with QuantiTect Reverse Transcription Kit (Qiagen) according to the manufacturer's protocol.

All the PCR primers used are listed in Table 4. Amplified products of PCR were electrophoresed in 1.2% agarose gels and visualized with ethidium bromide (Amresco) staining. The bands of PCR product from the neuroblastoma cell line BE(2)-M17 were cut and DNA was purified using the Gel Extraction Kit (Promega). Then the PCR products were cloned into the pGEM-T Easy vector (Promega) for sequence identification in an Applied Biosystems 3100 DNA sequencer.

**Table 4:** Primers used to amplify *DHRS4L1 *mRNA.

usage	Primer	Sequence (5'→3)
5'RACE (touchdown PCR)	UPA-F	CTAATACGACTCACTATAGGGCAAGCAGTGGTATCAACGCAGAGT (long) CTAATACGACTCACTATAGGGC (short)
	*DHRS4L1 *E10-R	GAGCACAGGAAAGACACGATGCCAAGAG
		
3'RACE (1st round of nest PCR)	*DHRS4L1 *outer F	GGATGGACAAGGAAAAAGAGG
	Adaptor 3'outer R	TACCGTCGTTCCACTAGTGATTT
		
3'RACE (2nd round of nest PCR)	*DHRS4L1 *inner F	TTAGGCGAGCCAGAGGATTCTCTT
	Adaptor 3' inner R	CGCGGATCCTCCACTAGTGATTTCACTATAGG
		
RT-PCR	*DHRS4L1 *E*a *F	CAAGCCCACCGTGGAGCTCATCTGA
	*DHRS4L1 *E10-R	GAGCACAGGAAAGACACGATGCCAAGAG
		
RT-PCR	*DHRS4L1 *E1 F	ATGCACAAGGCGCGGCTACGAG
	*DHRS4L1 *E10 R	GAGCACAGGAAAGACACGATGCCAAGAG
		
RT-PCR	β-actin F	AAATCGTGCGTGACATTAA
	β-actin R	CTCGTCATACTCCTGCTTG

## Abbreviations

*DHRS4*: dehydrogenase/reductase (SDR family) member 4; *DHRS4L1*: *DHRS4 like 1*; *DHRS4L2*: *DHRS4 like 2*; NRDR: NADP(H)-dependent retinol dehydrogenase/reductase; RACE: rapid amplification of cDNA ends; RT-PCR: reverse transcription- polymerase chain reaction; TSS: transcription start site.

## Authors' contributions

ZJS and QXZ conceived and designed this study. ZJS performed the RACE PCR, RT-PCR, collected and analyzed the bioinformatics data and drafted the manuscript. GFL, XHS, QL, RJW, and HBC participated in the experiment design and the data analysis. XYX and XXS carried out part of the sequence blasting. DYH is responsible for the design, organization and supervision of the whole study. QXZ and DYH provided funding for the research. All authors read and approved the final manuscript.
